# Antifungal and Insecticidal Potential of the Essential Oil from *Ocimum sanctum* L. against Dangerous Fungal and Insect Species and Its Safety for Non-Target Useful Soil Species *Eisenia fetida* (Savigny, 1826)

**DOI:** 10.3390/plants10102180

**Published:** 2021-10-14

**Authors:** Martin Žabka, Roman Pavela, Kateřina Kovaříková, Jan Tříska, Naděžda Vrchotová, Jan Bednář

**Affiliations:** 1 Crop Research Institute, Drnovská 507, 161 06 Prague, Czech Republic; pavela@vurv.cz (R.P.); kovarikova@vurv.cz (K.K.); 2 Laboratory of Metabolomics and Isotopic Analyses, Global Change Research Centre, Bělidla 986/4a, 603 00 Brno, Czech Republic; triska.j@czechglobe.cz (J.T.); vrchotova.n@czechglobe.cz (N.V.); bednar.j@czechglobe.cz (J.B.)

**Keywords:** pathogenic and toxigenic fungi, insect pests, mosquito vector, *Ocimum sanctum*, human infections, non-target species, GC/MS analysis

## Abstract

The antifungal and insecticidal effect of the essential oil from *Ocimum sanctum* L. was evaluated using a model set of harmful organisms hazardous for health and the economy. Toxigenic and plant pathogenic filamentous fungi, including causal agents of human infections, were chosen as exemplary fungal groups—*Fusarium verticillioides, Penicillium expansum* and *Aspergillus flavus*. *Spodoptera littoralis* (African cotton leafworm), *Culex quinquefasciatus* (Southern house mosquito), the lymphatic filariasis vector and potential Zika virus vector, and the common housefly, *Musca domestica* were chosen as model insects. Major and minor active substances were detected and quantified using GC/MS analysis. Environmental safety was verified using the non-target useful organism *Eisenia fetida*. Significant antifungal and insecticidal activity, as well as environmental safety, were confirmed. The essential oil showed the highest efficacy against *A. flavus* according to MIC50/90, and against *S. littoralis* larvae according to LD_50/90_. The monoterpenoid alcohol linalool, *t-*methyl cinnamate, and estragole as phenylpropanoids were detected as effective major components (85.4%). The essential oil from *Ocimum sanctum* L. was evaluated as universal and significantly efficient, providing a high potential for use in environmentally safe botanical pesticides.

## 1. Introduction

*Ocimum sanctum* L. is a legendary aromatic plant of the *Lamiaceae* family. It is an erect plant, 30–60 cm tall, used by humans for more than 3000 years. It is no surprise that precisely in the area of the Indian subcontinent, the birthplace of one of the most ancient and advanced civilizations, it is considered one of the most sacred plants and has been abundantly used in Ayurvedic medicine thanks to its numerous important properties. *O. sanctum* is considered irreplaceable in the above-indicated holistic approach to the care of the body and spirit, and is called “The Queen of Herbs”. Besides the regions of India, universal use of this plant was also known in ancient Greece and Rome as well as other regions [1,2]. Thanks to its content of many highly active substances, *O. sanctum* has been the subject of research focused on its important properties that have a practical use, including its antioxidant, chemoprotective, anti-inflammatory and hepatoprotective properties, as well as dozen other important medicinal properties [3]. *O. sanctum* is also a very significant plant for its antifungal and insecticidal properties [4,5,6]. This biological efficacy offers great potential for, among other things, especially for the environmentally friendly protection against harmful agents. The implementation of globally recognised ecological practices in agriculture and food production is closely linked to the use of non-synthetic pesticides, i.e., substances or products that are effective and acceptable for the environment. On the other hand, it is well known that the impossibility of using synthetic pesticides in modern organic farming still causes huge losses in terms of quantity and quality of the final products. [7,8,9]. Moreover, efforts to restrict the use of synthetic pesticides and thus reduce the environmental load have also been observed in conventional agricultural systems in recent years [10]. The study of natural and environmentally acceptable antifungal and insecticidal substances is an immense challenge for current scientific research [11,12,13,14,15]. This paper is primarily focused on the search for safe new alternatives that could be used for protection against hazardous toxigenic and pathogenic fungi and problematic insect pests, often vectors of dangerous human infections [12,16,17]. The antifungal and insecticidal effect of the essential oil (EO) from *O. sanctum* on toxigenic and pathogenic species of filamentous fungi that are important in both agriculture and medicine, specifically *Fusarium verticillioides*, *Penicillium expansum* and *Aspergillus flavus*, is determined, evaluated and described in this paper. Additionally, it studies the effects against important species of harmful and problematic insects, specifically *Spodoptera littoralis* (African cotton leafworm), *Culex quinquefasciatus* (Southern house mosquito), the lymphatic filariasis vector and potential Zika virus vector [18,19] and finally, the effects against the common housefly, *Musca domestica*. The paper also provides evidence of the environmental safety of the *O. sanctum* EO for non-target useful organisms, shown using the model soil organism (earthworm) *Eisenia fetida*. Major biologically active constituents of the EO were identified and quantified using GC/MS analysis.

## 2. Results and Discussion

The final yield of EO was 1.55 ± 0.02% (*w*/*w*, on a dry matter basis). The ten main identified components of *Ocimum sanctum* essential oil make up about 90%, approximately half of the whole EO belonging to the group of monoterpenoid alcohols (linalool). About the second half make up estragole and methyl cinnamate (phenylpropanoids) and eugenol (allylbenezene derivative) (Table 1). The dominant substances are linalool, estragole and *t-*methyl cinnamate, which together make up 85.49% of the total amount of compounds contained in the essential oil. In addition, other minority compounds (5.2%) such as eucalyptol, eugenol, *c*-methyl cinnamate, ocimene, terpinen-4-ol, bornyl acetate and camphor, which have been reported to possess strong biological activities, were detected as well. We suppose some of these minority compounds could be implicated in synergism. A total of 15 substances were analysed from which 10 compounds were identified. RI values were measured for all substances and the measured values fall well within the range of published indices [20]. To illustrate the possibility of identification of unknown compounds using retention indices (RI) we measured and compared three following substances for which standards were not available:
CompoundRI measuredRI data range from the literatureβ-Z-ocimene10481032–1061Bornyl acetate12961261–1297*c*-Methyl cinnamate13141301–1321

Babushok et al. [20] stated in their fundamental work on RI of terpenes in EO an average standard deviation value of 7.9 for the RI on dimethylsilicone stationary phase with 5% phenyl groups and a value of 25.5 for the averaged 90% confidence RI range.

**Figure 1:**
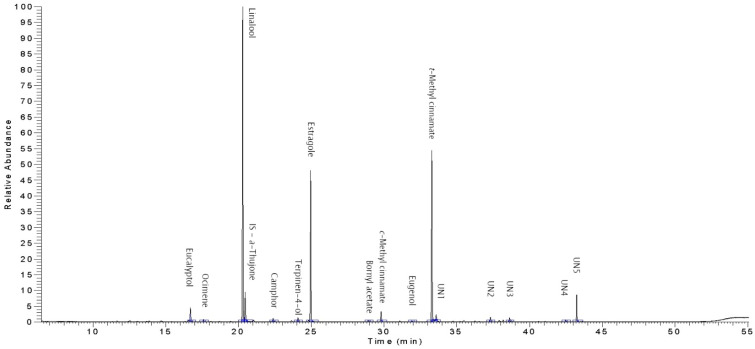
TIC chromatogram of the *Ocimum sanctum* essential oil.

All compounds were well resolved, as can be seen from the total ion current (TIC) chromatogram of *O. sanctum* EO (Figure 1). According the analyses, the *O. sanctum* studied by us cannot be assigned to the commonly reported eugenol chemotype [21], but rather to the linalool-estragole chemotype [22]. Eugenol is most often described in the literature as the main substance in the EO of *O. sanctum*. However, samples of *O. sanctum* with a higher content of linalool are mentioned as well. To some extent, the geographical location of cultivation may also contribute to the composition and content of the main components [23,24,25].

The essential oil from *O. sanctum* showed significant antifungal activity against the toxigenic and pathogenic filamentous fungi *F. verticillioides, P*. *expansum* and *A. flavus.* The highest inhibitory effect was observed for *A. flavus*, as follows from the lowest MIC_50/90_ values of 0.64/1.55 mg/mL, respectively. Similarly, with the statistically most significant difference, the essential oil from *O. sanctum* was efficient against *F. verticillioides*, with MIC_50/90_ values of 0.73/2.15 mg mL^−1^. This efficacy on *A. flavus* and *F. verticillioides* is significantly higher compared to *P. expansum*, where the MIC_50/90_ values of 1.51 and 4.9 mg mL^−1^ do not even overlap on the confidential interval (CI^95^) level. Paradoxically, for instance, *A. flavus* showed the highest resistance in our previous studies [26,27], which can be observed in this study, as well—as regards, for example, the effect of propiconazole used as a positive control. The MIC_50/90_ values of 1.43/31.3 mg L^−1^ are significantly higher for propiconazole against *A. flavus* compared to other fungi (Table 2).

We believe that the relatively high values of the inhibitory effect of the *O. sanctum* essential oil against target filamentous fungi may be due to the characteristically high content of the monoterpenoid alcohol linalool and of the phenylpropanoid estragole. Moreover, the GC/MS analysis confirmed a minor content of the extremely efficacious allylbenzene derivative eugenol. Eugenol is among the plant constituents having the highest antifungal activity [9,11,26]. Important antifungal activity has also been confirmed for linalool and estragole [28]. The antifungal effect of *O. sanctum* essential oil is lower compared to essential oils with high eugenol abundance, such as the essential oil from *P. dioica* [26,29]. Generally, it can be noted that components of the group of acyclic monoterpenoid alcohols such as linalool or the phenylpropanoid estragole do not achieve antifungal efficacy as high as that of eugenol or the even more efficacious thymol [11,12,30,31]. The last of the major substances, methyl cinnamate, is described as having a rather weak antifungal effect [32,33]. Nevertheless, based on a comparison of the MIC values of many other essential oils from previous studies [12,33], and GC/MS analysis, it can be noted that the essential oil from *O. sanctum* falls in the group that exhibits a high antifungal effect against filamentous fungi, primarily due to its high abundance of linalool and estragole. In some cases, however, linalool may have a higher antifungal effect than eugenol [34]. The mechanism of action of linalool and estragole has not been sufficiently explained. However, as in the case of eugenol, specific destabilization of the cell membrane function is induced, resulting in disturbed ion balance. Moreover, enzymes involved in pectin formation in the cell wall such as pectin methyl esterases are blocked, and the production of ergosterol is impaired thanks to ergosterol binding affinity. That results in the perforations or lethal deformations of the cell wall [11,35,36].

Toxicity against important insect pests, vectors of infectious diseases and problematic insects has been demonstrated for the essential oil from *O. sanctum* using the models of *Spodoptera littoralis*, *Culex quinquefasciatus* and *Musca domestica* (Table 2). As expected, in none of the cases was the toxicity statistically comparable to that of the pyrethrum extract used as a positive control. For *Spodoptera littoralis* larvae, significant contact toxicity was confirmed in an experiment with LD_50/90_ values of 39.3/74.5 µg larva^−1^. A lower efficacy was observed for *Musca domestica* adults. In this case, LD_50/90_ values reached 58.1/95.2 µg larva^−1^, but the toxicity difference apparent from the LD_50_ values is not statistically significant between both species, as indicated by the CI_95_ overlap (Table 2). The tests also showed larvicidal effect on the mosquito larvae of *Culex quinquefasciatus*, which is a vector of hazardous infectious diseases in humans [18,19]. In this species, the LD_50/90_ values were 89.5/120.6 mg L^−1^. The much higher effect on the mosquito larvae of *C. quinquefasciatus* (LD_50_ 26 mg L^−1^) is described in the case of *O. sanctum* essential oil purely extracted from leaves [37].

Although the main share of the detected major compounds with described insecticidal activity is represented by linalool, we believe that the larvicidal effect of *O. sanctum* oil on *Spodoptera littoralis* and *Culex quinquefasciatus* larvae is due instead to the content of *t*-methyl cinnamate. This substance exhibits a significantly higher larvicidal effect compared to linalool [38]. Linalool efficacy against *Musca domestica* adults has been confirmed in multiple studies; however, both linalool and *t-*methyl cinnamate are classified as moderate-efficacy substances among monoterpenoids and phenylpropanoids [39,40]. As regards another highly abundant substance, estragole, its insecticidal activity is lower compared to linalool [41]. The mechanism of the insecticidal effect of all three major substances targets predominantly the nervous system of the insects. Monoterpenoids alcohol such as linalool, in particular, cause strong inhibition of acetylcholinesterase (ACHe). An effect on gamma-aminobutyric acid (GABA) has also been described [42]. The effect on ACHe inhibition and GABA receptors may synergistically enhance the insecticidal activity. The safety of monoterpenoids for vertebrates, including humans, is ensured by morphological and molecular differences in GABA receptors, as well as by the differences between ACHe molecules within these groups of organisms [43]. Environmental safety of the essential oil from *O. sanctum* was verified using an experiment with the model earthworm *Eisenia fetida* (Table 3). No toxicity to this useful non-target soil species was observed even in high doses (up to 300 mg kg^−1^). Even a dose of 500 mg kg^−1^ showed significantly lower toxicity compared to α-cypermethrin in a concentration orders of magnitude lower, used as a positive control.

The significant fungicidal and insecticidal properties of the essential oil from *O. sanctum*, together with its confirmed environmental safety, reinforced by the abundant use of this sacred plant in folk medicine for millennia, indicate the great potential of this species for the subsequent research and development of safe products, including botanical fungicides and insecticides. Modern trends in the suppression of important agents that are harmful for health and the economy, such as toxigenic and pathogenic fungi or harmful hazardous insects, are dependent on the research into natural alternatives. Based on an overall comparison with the properties of other efficient essential oils [12,13,33] in terms of environmental safety and efficacy in the multi-species model spectrum of three important pathogenic filamentous fungi and three important representatives of harmful insects, it can be noted that the essential oil from *O. sanctum* provides sufficient biological activity and the potential capacity for universal use in the development of safe botanical preparations.

## 3. Materials and Methods

### 3.1. Plant Material and Essential Oil Isolation

*O. sanctum* plants were obtained from the experimental field of the Crop Research Institute (Prague, Czech Republic) where they were grown (GPS: 50.0864428N, 14.2985553E, soil type: illimerized luvisol, soil pH: 6.8, total annual precipitation: an average of 500.7 mm, Average annual temperature: 8.6 °C). The plants were harvested in the early stages of flowering in 2019. Plant material was in the form of aerial parts. Air-dried plants (30 g) of *O. sanctum* were manually reduced into small pieces, then inserted into a 1-L flask filled with 0.5 L of distilled water and subjected to hydrodistillation using a Clevenger-type apparatus for 3 h. Three replicates were used for hydrodistillation. The oil obtained was separated from the water and dried over anhydrous Na_2_SO_4_. The EO was stored in amber vials sealed with PTFE-silicone caps at +4 °C until the chemical analysis and biological assays.

### 3.2. Chemical Analysis

#### 3.2.1. Preparation of Distilled EO for Measurement

The essential oil obtained by hydro distillation were diluted with hexane (10 μL of EO to 990 μL of hexane) and 2 μL of this solution was finally diluted with 998 μL alpha-thujone solution—1.84 μg/mL (10 μL = 9.2 mg) was dissolved in 10 mL of hexane and then 200 µL of this solution was transferred into 100 mL of hexane). Each EO sample was thus prepared for measurement in triplicate.

#### 3.2.2. Qualitative and Quantitative Analysis of *O. sanctum* Essential oil by GC/MS

Terpenes of *Ocimum sanctum* EO was analysed on a Trace GC Ultra gas chromatograph (Thermo Fischer Scientific, San Jose, CA, USA) equipped with a Restek fused silica capillary column, Rxi-5 ms, 30 mm × 0.25 mm × 0.25 μm (Restek Corporation, Bellefonte, PA, USA), liner SKY, Splitless, 3 mm × 0.8 mm × 105 mm (Restek Corporation,) and coupled to a mass selective detector ISQ (Thermo Fischer Scientific) working at 70 eV of ionization energy. Helium was used as a carrier gas at 1.0 mL/min with injection of 1 μL in splitless mode at 250 °C. Split flow after 1 min 50 mL/min. The oven temperature was programmed as follows: 40 °C held for 5 min, then increased to 150 °C at a rate of 3 °C/min, then increased to 250 °C at a rate of 10 °C/min, then increased to 290 °C at a rate of 25 °C and finally maintained at 290 °C for 2 min. The temperature of the transfer line was maintained at 250 °C, and the temperature of the ion source was maintained at 200 °C. Scanning was performed after 7 min in the TIC mode in the range of 50–450 m/z. Component identification was made based on comparison of their mass spectra with the spectra of authentic standards, comparison of their retention data by co-injection of available standards with the exception of compounds denoted with an asterisk where the NIST library was used. Quantification was done by internal standard method using α-thujone, data after correction by response factor were expressed in percent obtained by ratio of corrected peak area to the total area of the peaks. The data are given in Table 1.

### 3.3. Target Organisms

#### 3.3.1. Fungal Strains

All target pathogenic and toxigenic fungal strains of *Fusarium verticillioides, Penicillium expansum* and *Aspergillus flavus* were obtained from the collection of phytopathogenic fungi maintained in the Crop Research Institute, v.v.i., Czech Republic, Prague. Strains were isolated originally from an infected corn cob and were identified by means of sequencing of ITS regions of the ribosomal DNA (rDNA). Strains were preserved on slant agar (potato carrot agar) at 4 °C. Subcultivations on Petri dishes and other manipulations with these strains were carried out in a Biosafety Level Two (BSL-2) laboratory, given the BSL of the *Fusarium* and *Aspergillus* species used in our experiment.

#### 3.3.2. Insect Rearing

The insect pest species tested in this study, namely *C. quinquefasciatus* larvae, *M. domestica* adults and *S. littoralis* larvae, were reared following the method recently reported by Benelli et al. (2019a) [44]. All species were obtained from an established laboratory colony (>20 generations) and maintained at 25 ± 1 °C, 70 ± 3% R.H. and 16:8 h (L:D).

### 3.4. Inhibitory Effect of O. sanctum on Target Filamentous Fungi and Experiment Design

The antifungal inhibitory effect of essential oil on mycelial radial growth of filamentous fungi was tested by the agar dilution method. *O. sanctum* essential oil was properly diluted in potato dextrose agar (PDA) in graded concentrations (0.1–4 mg/mL). The prepared Petri dishes (9.0 cm diameter) were aseptically inoculated with assay disc (0.4 cm) cuts from the periphery of a 7-day-old culture of the target fungi. The control sets were prepared subsequently using sterile distilled water instead of oil. The synthetic fungicide propiconazole (Sigma-Aldrich, Prague, Czech Republic; p.a.) was used as a positive control in graded concentrations (0.25–32 mg L^−1^). All experiments were performed in quadruplicates. The incubation was carried out at 21 °C for seven days. The percent inhibition of the radial growth of the target fungi was calculated according to the following formula: percent inhibition = (DC − DT)/DC × 100, where DC is the colony diameter of the control sets and DT is the colony diameter of the treated sets. The MIC_50_ was regarded as the concentration of plant extract that results in a 50% inhibition of visible growth when compared to control sets. The MIC_90_ was regarded as the lowest concentration of oil with 90% visible growth reduction when compared with control sets [11,26].

### 3.5. Insecticidal Activity of O. sanctum Essential Oil against Culex quinquefasciatus

25 individuals of 3rd instar larvae of *C. quinquefasciatus* were used for the bioassay in accordance with the methodology of the WHO (1996) [45], with minor changes by Pavela [46]. The variants evaluated were as follows: the essential oil (EO) diluted in dimethyl sulfoxide (DMSO) at concentrations of 20, 40, 60, 80, 100, 120, 150, 200, 250, 300, 400, 500 and 800 mg L^−1^, negative control (distilled water mixed with the same amount of DMSO as the EO variants) and positive control (pyrethrum extract 50%, Sigma-Aldrich, at concentrations of 0.02, 0.04, 0.06, 0.08 and 1.00 mg L^−1^). Each variant had four replicates and larval mortality was recorded after 24 h.

### 3.6. Insecticidal Activity of O. sanctum Essential Oil against Musca domestica

Twenty adult female individuals (3–6 days old) of *M. domestica* were used for the topical application in accordance with the methodology of Benelli et al. [44]. The tested variants (replicated four times) were as follows: *O. sanctum* (EO) diluted in 1 μL of acetone (Sigma-Aldrich, Taufkirchen, Germany) at concentrations as follows: 40, 60, 80, 100, 120, 150, 200, 250, 300, 350, 400, 450 and 500 µg per adult, negative control (acetone) and positive control (pyrethrum extract 50%, Sigma-Aldrich, Czech Republic, at concentrations of 2, 4, 6, 8 and 10 µg per adult). Flies were anesthetized using CO_2_ and the test substances were applied using a microelectric applicator to the pronotum of each individual. After the treatment, flies were moved to a recovery box (10 cm × 10 cm × 12 cm, 26 ± 1 °C 16:9 L:D). Mortality was recorded after 24 h [44].

### 3.7. Insecticidal Activity of O. sanctum Essential Oil against Spodoptera littoralis

Similar to the previous bioassay, topical application was used to evaluate the toxicity of the *O. sanctum* essential oil to *S. littoralis*. 20 individuals of 3^rd^ instar larvae of *S. littoralis* were used in each of the 4 replicates. The larvae were treated on the dorsum with 1 μL of acetone containing 20, 40, 60, 80, 100, 120, 140, 160, 180 and 200 µg of *O. sanctum* essential oil per larvae. Acetone was also used as the negative control. The positive control was pyrethrum extract (50%, Sigma-Aldrich, Czech Republic) tested at doses of 4, 8, 12, 16 and 20 µg per larvae. After the treatment, larvae were moved to a recovery box (10 cm × 10 cm × 7 cm, with air vents to avoid fumigation effects, 26 ± 1 °C, 70 ± 3% RH, and 16:8 L:D). Mortality was recorded after 24 h [44].

### 3.8. Toxicity of Ocimum Sanctum Essential Oil to Eisenia fetida (Non-Target Organism)

Adult earthworms *E. fetida* with well-developed clitella and weighing between 350–500 mg were obtained from a fixed laboratory colony (more than 20 generations; out-crossed once) following Pavela et al. (2018) [47] in the Crop Research Institute, Czech Republic. Ten individuals were used in each of the four replicates. The bioassay was done according to the OECD methodology (1984) [48]. Essential oil (emulsified with Tween 85, Sigma-Aldrich, Czech Republic) was added to the soil at 500, 300, 150, 100 and 50 mg kg^−1^. α-cypermethrin [Vaztak^®^ at 500 mg kg^−1^], diluted in water, at 10 mg kg^−1^ of dry soil was the positive control—following OECD methodology (1984) [48]. Distilled water was a negative control. Treated soil samples (650 g) were placed into one-litre glass jars, covered with gauze [49], and stored in a climate chamber (20 ± 1 °C, 80–85% RH, 16:8 L:D with 600 lux). Mortality was recorded 5 and 10 days after the treatment.

### 3.9. Statistical Analysis

#### 3.9.1. Antifungal and Insecticidal Assays

Abbott’s formula [50] was used to correct the data for control mortality, which should not exceed 20%. Therefore, the five best concentrations were selected from all tested concentrations. Then, using BioStat software (version 5, AnalystSoft Inc. Walnut, CA, USA), the estimation of insecticidal (LD_50;90_) and antifungal (MIC_50;90_) values was done by analysis of binomial response variables (Probit analysis) [51]. The obtained LD and MIC values were associated with a 95% confidence interval and Chi-square values significant at the *p* < 0.05 level.

#### 3.9.2. Toxicity to Non-Target Soil Organisms

Statistica software (version 13.3, Tibco, Palo Alto, CA, USA) was used for statistical analysis. Before running ANOVA, the data were adjusted by the arcsine square root transformation (arcsine√). Differences between the variants were determined by Tukey’s test (*p* ≤ 0.01).

## Figures and Tables

**Table 1:** Qualitative and quantitative analysis of *Ocimum sanctum* essential oil by GC-MS.

Compound Class	EO Component	RI	Normalized Area [%]	SD
Monoterpenoid, hydrocarbon	Ocimene *	1048	0.5	0.01
*Monoterpenoid, ketone*	*IS*—*α*-Thujone	*1108*	*x*	*x*
Monoterpenoid, bicyclic ether	Eucalyptol	1030	1.7	0.03
Monoterpenoid, alcohol	Linalool	1105	42.3	0.59
Monoterpenoid, bicyclic ketone	Camphor	1145	0.3	0.01
Monoterpenoid, alcohol	Terpinen-4-ol	1178	0.8	0.01
Acetate ester of borneol	Bornyl acetate *	1296	0.3	0.01
Phenylpropanoid	Estragole	1194	22.9	0.16
Phenylpropanoid	*c*-Methyl cinnamate *	1314	1.4	0.03
Phenylpropanoid	*t*-Methyl cinnamate	1382	19.9	0.47
Allylbenzene deriv.	Eugenol	1355	0.5	0.03
Unidentified components	UN1	1388	1.5	0.05
	UN2	1479	1.9	0.01
	UN3	1513	1.1	0.02
	UN4	1599	0.5	0.01
	UN5	1598	4.5	0.08

Component identification was performed based on comparison of their mass spectra with the spectra of authentic standards, with the exception of compounds denoted with the asterisk (*).

**Table 2:** Antifungal and insecticidal activity of the essential oil from the *Ocimum sanctum* against target species.

Target Species			MIC_50_/LC (LD)_50_	CI_95_	MIC_90_/LC (LD)_90_	CI_95_	*Chi* ^a^	*p*–Value
*F. verticillioides*	*EO*	(mg mL^–1^)	0.73 ± 0.04	0.64–0.82	2.15 ± 0.24	1.78–2.78	0.242	0.886 ns
	propiconazole	(mg L^–1^)	0.68 ± 0.09	0.51–0.87	11.8 ± 2.2	8.49–18.04	0.455	0.998 ns
*P. expansum*	*EO*	(mg mL^–1^)	1.51 ± 0.13	1.31–1.83	4.9 ± 0.95	3.62–7.72	0.212	0.899 ns
	propiconazole	(mg L^–1^)	0.41 ± 0.04	0.33–0.51	2.51 ± 0.31	2.02–3.29	0.451	0.998 ns
*A. flavus*	*EO*	(mg mL^–1^)	0.64 ± 0.04	0.55–0.72	1.55 ± 0.16	1.34–1.89	1.17	0.557 ns
	propiconazole	(mg L^–1^)	1.43 ± 0.18	1.11–1.81	31.3 ± 3.06	20.8–53.4	0.345	0.998 ns
*Cx. quinquefasciatus* larvae	*EO*	(mg L^–1^)	89.5 ± 3.3	78.8–108.8	120.6 ± 11.1	109.1–137.1	6.505	0.164 ns
	pyrethrum	(mg L^–1^)	0.02 ± 0.00	0.01–0.03	0.05 ± 0.01	0.04–0.06	1.231	0.578 ns
*M. domestica* adults	*EO*	(µg adult^–1^)	58.1 ± 3.6	33.9–66.7	95.2 ± 2.1	83.2–109.7	2.602	0.271 ns
	pyrethrum	(µg adult^–1^)	0.18 ± 0.2	0.17–0.21	0.91 ± 0.1	0.82–1.15	1.538	0.597 ns
*S. littoralis* larvae	*EO*	(µg larva^–1^)	39.3 ± 2.5	28.2–44.7	74.5 ± 6.4	62.4–81.4	0.789	0.837 ns
	pyrethrum	(µg larva^–1^)	0.1 ± 0.05	0.1–0.2	1.5 ± 0.3	1.3–1.8	1.562	0.652 ns

Minimum inhibitory concentration (MIC_50_ and MIC_90_) and lethal concentration (LC_50_ and LC_90_) or lethal doses (LD) values and CI_95_—95% confidence intervals, essential oil activity is considered significantly different when the 95% CI fail to overlap. ^a^ Chi-square values: not significant (*p* > 0.05).

**Table 3:** Lack of toxicity of *O. sanctum* essential oil on non-target *Eisenia fetida* earthworms.

Essential Oil (Dose mg·kg^−1^)	Mortality (%) ^a^ ± SE	
	7th Day	14th Day
500	10.0 ± 5.0 b	10.0 ± 5.0 b
300	0.0 ± 0.0 a	0.0 ± 0.0 a
150	0.0 ± 0.0 a	0.0 ± 0.0 a
100	0.0 ± 0.0 a	0.0 ± 0.0 a
50	0.0 ± 0.0 a	0.0 ± 0.0 a
Negative control ^b^	0.0 ± 0.0 a	0.0 ± 0.0 a
Positive control (α-cypermethrin 0.1 mg.kg^−1^)	85.0 ± 5.0 c	100.0 ± 0.0 c
ANOVA *F_6,21_*, *p*-value	423.5, <0.001	1329.0, <0.001

^a^ Average mortality of *E. fetida* (± SE) achieved on the 7th and 14th day after application of EOs; within a column, means ± SD followed by the same letter do not differ significantly (Tukey’s HSD test).% = arcsine square root transformed data. ^b^ Negative control = distilled water + Tween 80 (200 mg kg^−1^).

## Data Availability

Not applicable.
